# Hemorrhoid Energy Therapy for Treatment of Deep Chronic Anal Fissures

**DOI:** 10.14309/crj.0000000000000172

**Published:** 2019-08-23

**Authors:** Fahad Malik, Jonathan Reyes, Bhavin Patel, Marina Kim, Manuel Gonzalez, Prasanna Wickremsinghe

**Affiliations:** 1 Internal Medicine, Richmond University Medical Center, Staten Island, NY; 2 American University of Antigua, New York Headquarters, New York, NY; 3 Division of Gastroenterology, New York Presbyterian Brooklyn Methodist Hospital Weill Cornell College of Medicine, Brooklyn, NY

## Abstract

Energy therapy is a well-known, minimally invasive treatment for internal hemorrhoids. This treatment has not yet been studied in anal fissures refractory to medical therapy. Anal fissures, such as hemorrhoids, can disrupt the quality of life for patients. Currently, nonsurgical treatments available for anal fissures are only supportive measures. Definitive for chronic anal fissures refractory to medical therapy is internal sphincterotomy. This treatment has chances of relapse and a risk of anal incontinence. We propose the use of hemorrhoid energy therapy with bipolar cautery as a safer, less invasive, and effective therapy for recurrent anal fissures refractory to conservative management.

## INTRODUCTION

Anal fissures are anal ulcerations due to repetitive microtrauma, usually due to passage of hard stools. These are considered to be chronic anal fissures (CAFs) if persistent beyond 6 weeks. Anal fissures, such as hemorrhoids, can severely disrupt the quality of life for patients. The gold standard for CAFs refractory to medical therapy is treatment with internal sphincterotomy. Unfortunately, with this treatment, there is a chance of relapse and a risk of anal incontinence. Nonsurgical measures are supportive treatments which usually include increasing dietary fiber intake, stool softeners, sitz baths, topical analgesics, or vasodilators. Energy therapy is a well-known treatment option for symptomatic Grades I and II internal hemorrhoids, but this treatment has not yet been studied in anal fissures refractory to medical therapy. We propose the use of hemorrhoid energy therapy with bipolar cautery as a safer, less invasive, and effective therapy for recurrent anal fissures refractory to conservative management.

## CASE REPORT

A 53-year-old obese heterosexual man with a medical history of Type II diabetes, prostatitis, constipation, recurrent anorectal fissures for the past 3 years, internal hemorrhoids, and intermittent rectal bleeding after transrectal prostate biopsy 3 years ago presented to our hospital with bright red blood per rectum and severe constant perianal pain for 8 hours exacerbated by sitting and straining during bowel movements. He had this pain for 2 months, which now acutely worsened. He had been managed outpatient for chronic constipation with stool softeners and laxatives (docusate sodium 100 mg at bedtime, bisacodyl 10 mg daily, and polyethylene glycol 17 g 3 times a day) as well as topical analgesics (2% lidocaine ointment as needed) and vasodilator ointment (0.2% nitroglycerin ointment) for the treatment of his painful anal fissure. Rectal examination revealed a large clot and anal tenderness. No external hemorrhoids were seen and no mass was felt. His hemoglobin was 13.5 g/dL on admission and dropped to 10.5 g/dL in about 14 hours because of continued bleeding per rectum, with likely some degree of hemodilution after administration of intravenous fluids.

As the patient was hemodynamically stable, urgent deep sedation colonoscopy with propofol performed within 24 hours revealed 3 internal nonbleeding hemorrhoids located at the 4-, 7-, and 11-o'clock positions and a slowly oozing bleeding anterior anorectal fissure. The anal fissure exhibited characteristics of chronicity with base being exposed to the internal anal sphincter, hypertrophic anal papilla, and sentinel pile located on the dentate line at the 7-o'clock position. Four cc of 1:10,000 epinephrine was injected proximal to the fissure with adequate cessation of bleeding. Hemorrhoid energy therapy with bipolar cautery was applied to the bleeding anal fissure using the same technique as one would apply for hemorrhoid treatment, and adequate cessation of bleeding was achieved. The tissue consisting of the anal fissure in the bowel wall was compressed in a parallel fashion, and bipolar radiofrequency energy was delivered until the temperature reached 55°C or 131°F (5–20 seconds) for approximately 1.5–2 seconds. In addition, all 3 internal nonbleeding hemorrhoids were also rectally ablated using the same Medtronic hemorrhoid energy therapy hemorrhoid instrument, and no further bleeding was detected. The patient did not require any analgesics after the procedure and showed significant improvement with no further episodes of hematochezia or rectal pain within a day.

## DISCUSSION

Anal fissures, also known as anal ulcers, are typically caused by microtrauma to the squamous epithelium within the distal portion of the anal canal, usually due to hard stool. These usually occur because of repetitive microtrauma or increased pressure, resulting in a longer duration of healing process. Primary causes are usually because of local trauma from diarrhea, constipation, vaginal delivery, or anal sex, which lead to the tearing of the mucosa or fissures to develop. Secondary causes have been linked to conditions such as Crohn's disease, other granulomatous diseases, malignancy, and infectious diseases such as HIV, syphilis, and chlamydia.^1,2^

Primary management is usually with supportive measures, such as increase in fiber, sitz bath, topical analgesics and use of topical vasodilators like nifedipine or nitroglycerin. The definitive surgical treatment for anal fissures refractory to conservative management is internal sphincterotomy, which reduces the resting anal pressure allowing for healing and aids in preventing future development of fissures. However, there is a risk of relapse and/or a risk of anal incontinence.^3–6^ In one study, the observational study reoccurrence rate of fissure was 11.6% and complication of anal incontinence was 2.3%.^7^ In review, sequelae of 829 patients reported a lack of control on flatus in 35.1% and soiling of clothing in up to 22%.^6^ This can occur in up to 45% of patients immediately postoperatively; however, it improves in the long term for most patients.^1,5,8^ We propose a novel alternative treatment for anal fissures refractory to conservative medical management that is safe and effective.

The exact mechanism of healing after bipolar cautery treatment for CAFs is still uncertain. Hemorrhoid energy therapy uses temperature to decrease blood supply of the hemorrhoid. We believe that a similar mechanism can effectively help heal fissures. The delivery of energy to a targeted region with temperatures between 50°C and 55°C (122°F–131°F) along with compression of the tissue can help obtain homeostasis for bleeding or CAF along with the change of histology to mild scarring with fibrosis which would result in healing of the fissure.^9^ Other possible mechanisms could involve removal of irritancy from associated hemorrhoids, alteration of blood flow to the anorectal region, increasing inflammation to assist healing or decrease spasms of the internal sphincter muscle.^9–12^

This intervention to use bipolar cautery in patients with CAF-associated internal hemorrhoids was studied in a prospective study, with results showing complete healing of CAF in 9 of 10 patients. However, 90% of these patients had full recovery of CAF in 4 weeks with no reoccurrences or complications, it was a well-tolerated procedure by the patients, and it was cost effective and decreased the average time taken off from work in comparison with surgical intervention. One of the 10 patients developed a perianal abscess complicated by a fistula.^10,11^ Our patient was followed up 2 years after the procedure with no recurrences of anal fissures or rectal bleeding and reported improved quality of life with no complications from the procedure. As more clinical studies and data emerge, we can better assess hemorrhoid energy therapy for the treatment of CAF as an alternative therapy.

## DISCLOSURES

Author contributions: F. Malik, J. Reyes, and B. Patel wrote and edited the manuscript. M. Kim edited the manuscript. M. Gonzalez and P. Wickremsighe edited the manuscript. F. Malik is the article guarantor.

Financial disclosure: None to report.

Informed consent was obtained for this case report.
